# Ominous Occurrence of Spinal Intradural Primary Malignant Peripheral Nerve Sheath Tumor Four Decades following Radiation Therapy for Testicular Seminoma

**DOI:** 10.1155/2020/1792582

**Published:** 2020-01-27

**Authors:** Osmond C. Wu, Berje H. Shammassian, Arunit J. S. Chugh, Aparna Harbhajanka, Manish K. Kasliwal

**Affiliations:** ^1^Department of Neurological Surgery, University Hospitals Cleveland Medical Center, Cleveland, OH, USA; ^2^Department of Pathology, University Hospitals Cleveland Medical Center, Cleveland, OH, USA

## Abstract

Primary intradural malignant peripheral nerve sheath tumor (MPNST) is an extremely rare diagnosis and is associated with an extremely poor prognosis. A 77-year-old man diagnosed with an intradural MPNST, more than 40 years after radiation for a testicular seminoma, is reported. Intradural MPNSTs of the spine outside the setting of neurofibromatosis is extremely rare and can masquerade as common benign nerve sheath tumors, on imaging. An older age at presentation with short duration of symptoms and prior regional radiation treatment encompassing the spine in the treatment field regardless of remoteness should alert the oncologists and neurosurgeons to the possible existence of this rare and aggressive tumor, as the management, and overall prognosis of this tumor is distinctly different compared to the usual intradural spinal tumors.

## 1. Introduction

Malignant peripheral nerve sheath tumors (MPNSTs) are aggressive, locally invasive rare soft tissue sarcomas arising from peripheral nerves that originate from Schwann cells or pluripotent cells of neural crest origin [1, 2]. MPNSTs represent 3–10% of all soft tissue sarcomas, with an overall incidence of 0.001% in the general population that peaks in the seventh decade [2–4]. In patients with neurofibromatosis type 1 (NF1), the annual incidence is 1.6 per 1000 with a lifetime risk of 8–13% [5]. Prior radiation exposure is another important risk factor, with a reported incidence of radiation induced MPNST ranging from 5.5–11% of patients [6]. MPNSTs are usually located in the extremities, trunk, and head, and neck. Spinal MPNSTs, however, are exceedingly rare.  Table 1 comprises a list of the spinal MPNSTs in the literature. While there are a few instances of spinal and paraspinal MPNST following radiation for testicular seminomas, all the cases reported occurred within 7–10 years following radiation and were mainly extradural in location [7–10]. An intradural spinal MPNST with subsequent intracranial leptomeningeal metastasis diagnosed forty years after radiation is presented in this report with a pertinent review of the literature.

## 2. Case Report

### 2.1. History and Presentation

A 77-year-old man with past medical history significant for esophageal adenocarcinoma as well as testicular seminoma that was treated with conventional external bean radiation therapy (EBRT) almost 40 years back presented with three months of left hip and buttock pain in addition to left foot weakness. He had no history or clinical stigmata of neurofibromatosis.

**Figure 1:**
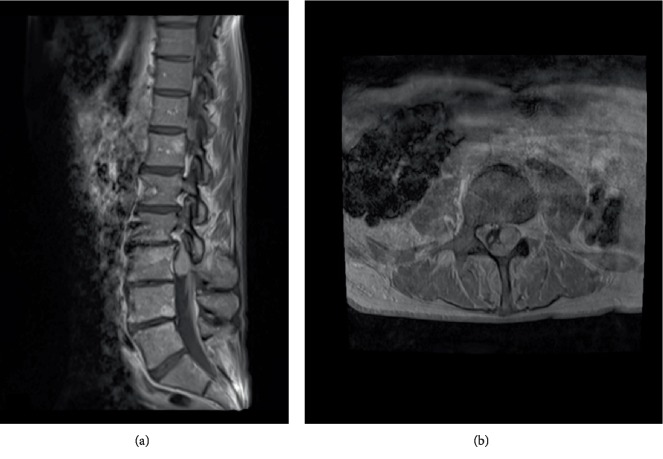
(a) Magnetic resonance imaging (MRI) T1-weighted sagittal image of the lumbar spine with gadolinium demonstrates a well demarcated intradural extramedullary mass. (b) Corresponding axial image.

A magnetic resonance imaging (MRI) of the lumbar spine with and without gadolinium demonstrated a homogenously enhancing, well demarcated intradural extramedullary neoplasm (Figure 1). An initial MRI of the cervical and thoracic spine with and without gadolinium was negative for additional lesions. While a diagnosis of benign nerve sheath tumor was suspected given typical radiological appearance, a metastatic lesion was also considered in the differential because of prior history of cancer. A metastatic work up performed to look for any additional lesions was negative.

### 2.2. Operative Course

**Figure 2:**
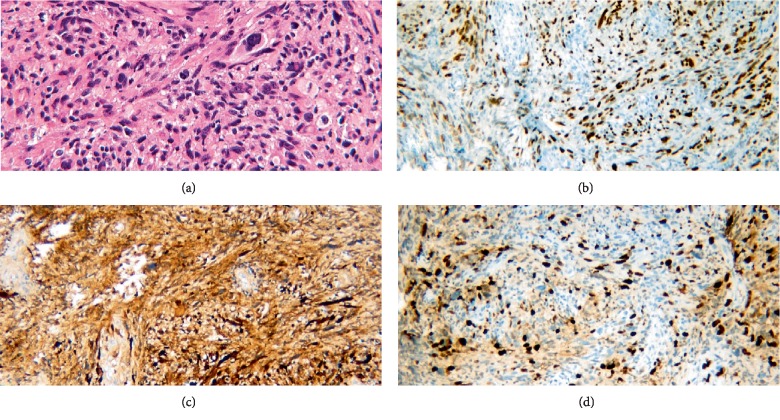
(a) Hematoxylin and eosin (H&E) staining showed high cellular density with marked pleomorphism and spindle cells arranged in fascicles (200x). (b) Immunohistochemistry was positive for SOX 10 (100x) and (c) S-100 (100x). (d) Ki-67 labeling revealed a high proliferative index (100x).

A partial L2 and complete L3 laminectomy was performed and a midline durotomy was made. A greyish mass was found in the intradural space arising from the left L3 nerve root with obvious enlargement and involvement of the nerve root. A frozen specimen was sent early for pathological evaluation that was diagnosed as MPNST. The nerve root of interest was clearly identified both proximally and distally, which on stimulation resulted in robust electrophysiological response suggestive of origin from a motor nerve root. No obvious plane was found between the tumor and the nerve root. Given the risk of motor deficit, the nerve root was preserved with partial resection of the tumor. Final pathology confirmed the diagnosis of MPNST (Figure 2).

### 2.3. Post-Operative Course

**Figure 3:**
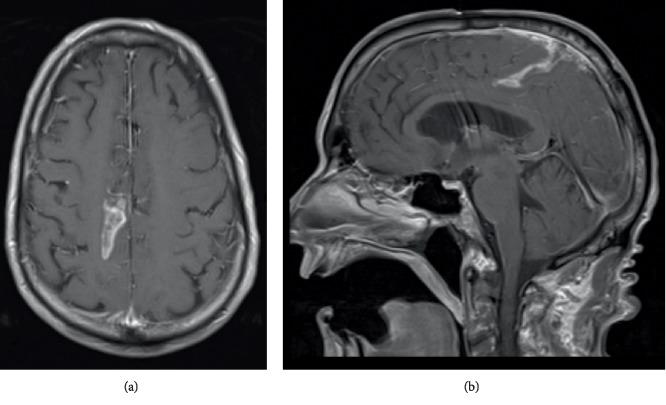
(a) Magnetic resonance imaging (MRI) T1-weighted axial image of the brain with gadolinium demonstrates leptomeningeal metastasis. (b) Corresponding sagittal image.

Given the initial pathologic diagnosis on frozen section, subsequent management including reoperation with extensive resection was discussed. Given his preoperative status, the patient, and family elected to observe in the short term with consideration of further treatment options following final pathology. The patient did well initially and was discharged to a skilled nursing facility. Two weeks post-operatively, however, the patient was readmitted with encephalopathy. An MRI brain with and without gadolinium demonstrated leptomeningeal metastasis (Figure 3) with cerebral spinal fluid (CSF) cytology positive for malignant cells. Given the extent of disseminated disease and his progressive worsening mental status, the family elected to pursue palliative care, and the patient died two months after his initial surgery.

## 3. Discussion

MPNSTs are rare entities with an incidence of 0.001%, with 20–50% of cases arising in patients with NF-1. The most common locations include the trunk, extremities, and head, and neck [2]. Primary spinal MPNSTs are extremely rare. Primary MPNSTs of the spine that are exclusively intradural extramedullary without extension into the extradural compartment are exceptionally rare. Outside the setting of NF-1, prior radiation treatment is a risk factor for development of MPNSTs. There have been few reports of MPNST following prior radiation for testicular seminomas. Most occurred after a short latent period and in an extradural location, with a purely intradural occurrence being exceptionally rare [7–10].

The present case developed almost four decades after being treated with EBRT for testicular seminoma, which is a significantly longer latency period as compared to other cases previously reported. Also, the imaging features were fairly characteristic of a benign intradural extramedullary neoplasm unlike a MPNST, which is typically an irregularly-bordered heterogeneously enhancing mass often with destruction of surrounding osseous structures. Similarly, the presence of leptomeningeal spread is also rare in primary intradural MPNSTs and as illustrated in this case, portends an ominous prognosis [11]. Unique to this case, however, is the rapid development of disseminated disease two weeks after surgery. Patients in other reported cases of intradural MPNSTs with leptomeningeal spread were diagnosed with dissemination ranging from present on presentation to 24 months following initial surgery [10–19]. The authors advocate that regardless of imaging characteristics or the duration since radiation, surgeons should retain a high index of suspicion for a MPSNT. Lumbar puncture may be considered to obtain CSF to identify potential malignant cells.

## 4. Conclusion

A rare case of an intradural MPNST diagnosed more than 40 years after radiation for a testicular seminoma is reported. Intradural MPNSTs of the spine outside the setting of neurofibromatosis are extremely rare and can masquerade common benign nerve sheath tumors on imaging. Short duration of symptoms and prior regional radiation treatment encompassing the spine in the treatment field regardless of remoteness should alert the physician to the possible existence of this rare and aggressive tumor. Being cognizant of this rare pathology can help initiate appropriate work up and evaluation, allow preoperative counselling, and alter overall surgical strategy.

## Figures and Tables

**Table 1:** Primary spinal malignant peripheral nerve sheath tumors (MPNST) reported in the literature.

Author & year	Age (yrs), sex	Location	Presentation	NF history	Radiation history	Extent of resection	Radiation therapy	Chemotherapy	Recurrence	Metastasis	Outcome
Thomeer et al., 1981 [12]	42, M	Cauda equina	Low back pain for 9 yrs with occasional L radiculopathy, impotence for 4 wks, leg weakness for 3 wks		Not specified	Total	T11-S4 2.5 Gy 4x/wk for 6 wks		Yes, 3 yrs T9-11	No	Alive at 3 yrs
Valdueza et al., 1991 [13]	40, F	Thoracic	Thoracic pain, R leg weakness		Not specified	Partial	Total 12 Gy after initial operation, total 40 Gy after spinal metastasis	No	Yes, 2 mo	Chest, pelvis, spine	Alive at 4 yrs
	43, F	Thoracic	Low back pain for 1 mo, leg weakness for 2 wks		Not specified	Partial	Total 24 Gy after first operation, total 32 Gy after second operation for recurrence	No	Yes, 8 yrs	No	Alive at 10 yrs
	47, M	Cervical	Neck pain radiating to R shoulder for 9 mo, R arm weakness for 1 mo	NF1	Not specified	Total	Total 10 Gy	No	Yes, 3 mos	Brain, lumbar	Dead at 18 mos
	18, M	Cervical	*L* shoulder and arm pain for 4 mos		Not specified	Total	No	No	No	No	Alive at 8 mos
	70, F	Cervical	Neck pain radiating to R shoulder for 6 mos		Not specified	Total	No	No	No	No	Alive at 7 mos
Seppälä et al., 1993	13, M	Lumbar	Paraparesis	NF1	12 yrs ago for Wilm's tumor	Partial	Not specified	Not specified	Yes	Brain, systemic	Dead at 2 mos
	13, M	Lumbar	Paraparesis		Not specified	Total	Not specified	Not specified	Yes	Systemic	Dead at 7 mos
	45, F	Sacral	Not specified	NF1	Not specified	Partial	Not specified	Not specified	Yes	Systemic	Dead at 2 mos
	35, M	Lumbar	Radiculopathy	NF2	Not specified	Total	Not specified	Not specified	Yes	Systemic	Dead at 18 mos
	23, F	Thoracic	Not specified		Not specified	Total	Not specified	Not specified	Yes	Systemic	Dead at 8 mos
	37, F	Cervical	Radiculopathy		Not specified	Total	Yes	No	Yes, 2 yrs	Systemic	Dead at 6 yrs
Celli et al., 1995 [15]	52, F	Thoracic	Pain for 8 mos, weakness		Not specified	Total	No	No	No	No	Alive at 6 yrs
	68, F	Lumbar	Pain for 9 mos, weakness		Not specified	Total	No	No	No	No	Alive at 2 yrs
	43, M	Lumbar	Pain for 3 mos		Not specified	Total	No	No	No	No	Alive at 6 yrs
	36, F	Thoracic	Pain for 5 mos		Not specified	Total	Yes	No	Yes	No	Alive at 4 yrs
	22, F	Cervical	Pain for 2 yrs, weakess, incontinence	NF1	Not specified	Total	Yes	No	Yes	Lung	Dead at 6 mos
	30, M	Thoracic	Pain for 3 yrs, weakness		Not specified	Total	Yes	No	Yes	Lung	Dead at 14 mos
West et al., 1997 [7]	40, M	Sacral	Radiculopathy		8 yrs ago for testicular seminoma, 30.6 Gy	Total	No	No	Yes, 7 mos	No	Not specified
Kourea et al., 1998 [8]	11, F	Lumbar, sacral	Not specified	NF1	Not specified	Partial	Yes	Yes	Yes, 0.5 mos	Yes, location not specified	Dead at 5 mos
	25, F	Lumbar	Not specified	NF1	Not specified	Partial	Yes	Yes	No	No	Alive at 18 yrs
	33, F	Lumbar	Not specified	NF1	Not specified	Partial	No	No	No	Yes, location not specified	Dead at 2 mos
	33, M	Lumbar	Not specified	NF1	Not specified	Partial	Yes	Yes	Yes, 3 mos	Yes, location not specified	Dead at 22 mos
	31, F	Lumbar	Not specified	NF1	Not specified	partial	yes	Yes	yes, 6 mos	yes, location not specified	dead at 10 mos
	37, F	Lumbar	Incidental		Not specified	Partial	No	Yes	Yes, 3 mos	No	Dead at 4 mos
	40, M	Sacral	Not specified		For testicular seminoma	Total	No	No	Yes, 9 mos	Yes, location not specified	Alive at 14 mos
	17, M	Thoracic	Not specified	NF1	Not specified	Partial	No	Yes	Yes	Yes, location not specified	Dead at 11 mos
	19, F	Thoracic	Incidental	NF1	Not specified	Total	No	No	No	No	Alive at 35 mos
	53, F	Thoracic	Not specified		For breast carcinoma	Partial	No	No	Yes, 6 mos	Yes, location not specified	Dead at 7 mo
	26, M	Thoracic	Not specified		For Hodgkin's lymphoma	Partial	Yes	No	Yes, 13 mos	Yes, location not specified	Dead at 27 mo
Acharya et al., 2001 [20]	32, M	Cauda equina	Back pain, leg weakness, bowel and bladder dysfunction		Not specified	Partial	Yes	No	No	No	Alive at 18 mos
Yone et al., 2004 [16]	4, M	Cauda equina	Low back pain, radiculopathy, bladder dysfunction		Not specified	Total	Yes	Yes	Yes	Brain, spine	Dead at 21 mos
Adamson et al., 2004 [17]	37, M	Cervical	L C6 radiculopathy		6 yrs ago for Hodgkin's lymphoma	Partial	Yes	No	Not specified	No	Dead after 1 yr
	30, F	Cervical	R Horner syndrome, R C6 radiculopathy, R tricep and wrist extensor weakness		5 yrs ago for Hodgkin's lymphoma	Partial	No	No	Not specified	No	Dead after 1 yr
Amin et al., 2004 [9]	38, M	Cauda equina	Back pain, leg weakness, bowel and bladder dysfunction		10 yrs ago for testicular seminoma, 30 Gy/15 fractions over 3 wks	Biopsy	No	Yes, palliative	Yes, 7 mos	Not specified	Not specified
Albayrak et al., 2006 [18]	25, M	Thoracic	Paraparesis, bladder dysfunction	NF1	Not specified	Total	No	No	Yes, 7 wks	Lung	Alive at 7 wks
Chamoun et al., 2009 [14]	5, F	Cervical	Pain, gait disturbance		Not specified	Partial	Yes	Yes	Yes	Brain, thoracic and lumbar spine	Alive at 4 mos
Xu et al., 2012	8, M	Lumbar	Pain		Not specified	Total	Yes	No	Yes	Brain	Dead at 16 mos
Mitsuhara et al., 2013	47, F	Cauda equina	Back pain, R leg weakness, bowel and bladder dysfunction, altered mental status	NF2	15 yrs ago for uterine and cervical cancer, 22 Gy	Partial	Yes, 36 Gy brain and spine, additional 14.4 Gy to lumbosacral lesion/28 fractions	No	Not specified	No	Not specified
Stark et al., 2013	56, F	Sacral	L leg radiculopathy, L foot paresis		15 yrs ago for non-Hodgkin's lymphoma	Not specified	No	Yes	Yes	Brainstem, spine	Dead at 24 mos
Wu et al., 2014	9, F	Thoracic, lumbar, sacral	R hip pain, bilateral leg weakness	NF2	Not specified	Partial	No	No	Yes	Brain	Dead at 9 mos
Li et al., 2014	33, F	Low thoracic, upper lumbar	Low back pain, R leg radiculopathy, hydrocephalus		Not specified	Partial	Yes, 28 Gy/19 fractions	No	Yes	Brain, diffuse spine	Dead at 29 mos
Lau et al., 2014 [10]	43, M	Cauda equina	Low back pain, L leg radiculopathy for 5 mos		10 yrs ago for testicular seminoma	Total	No	Yes, alternating between ifosfamide/doxorubicin and ifosfamide/etoposide	Yes, about 60 mos	Brainstem, cervical spine, renal	Dead at 5 yrs
Thomas et al., 2014	49, M	Cauda equina	Low back pain, paraparesis		Not specified	Partial	No	No	No	Brain and spine	Not specified
Baharvahdat et al., 2016	3, F	Cervical, upper thoracic	Paraplegia, bladder and bowel dysfunction		Not specified	Partial	No	No	No	Brain and spine	Dead shortly after surgery
Chou et al., 2017^a^	5–74 (mean 40)						76% had adjuvant therapy		38% over 2 yrs		59% alive at 2 yrs
Samancia et al., 2017	27, M	Cervical, upper thoracic	Hydrocephalus		Not specified	Partial	Yes	Yes	No	No	Not specified
Present study	77, M	Lumbar	Left hip and buttock pain for 3 mos		About 45 yrs ago for testicular cancer	Partial	No	No	No	Intracranial leptomeningeal disease	Dead at 2 mos

F: Female, Gy: gray, L: left, M: male, mos: months, NF1: neurofibromatosis type 1, NF2: neurofibromatosis type 2, R: right, wks: weeks, yrs: years.

^a^ Multicenter series (*N* = 29), individual patient data not available.

## Abbreviations

CSF: Cerebral spinal fluid
CT: Computer tomography
EBRT: External bean radiation therapy
H&E: Hematoxylin and eosin
MPNST: Malignant peripheral nerve sheath tumor
MRI: Magnetic resonance imaging
NF1: Neurofibromatosis type 1.
